# How does market competition affect supplier-induced demand? An experimental study

**DOI:** 10.3389/fpubh.2023.1024337

**Published:** 2023-03-08

**Authors:** Yefeng Chen, Yiwen Pan, Yuli Ding

**Affiliations:** ^1^School of Economics, College of Economics and Interdisciplinary Center for Social Sciences, Zhejiang University, Hangzhou, China; ^2^School of Economics, Zhejiang Gongshang University, Hangzhou, China

**Keywords:** medical market, market competition, supplier-induced demand, credence goods, economic experiment

## Abstract

**Introduction:**

This study investigated the impact of competition on supplier-induced demand in medical markets theoretically and experimentally.

**Methods:**

We employed the framework of credence goods to describe the information asymmetry between physicians and patients, and theoretically derives predictions of physicians' behaviors in monopolistic and competitive markets. Then we conducted behavioral experiments to empirically test the hypotheses.

**Results:**

The theoretical analysis revealed that an honest equilibrium would not exist in a monopolistic market, whereas price competition could induce physicians to reveal their types of treatment cost and provide honest treatments; thus, a competitive equilibrium is superior to that of a monopolistic market. The experimental results only partially supported the theoretical predictions, which showed that the cure rate of patients in a competitive environment was higher than that in a monopolistic market, although supplier-induced demand occurred more frequently. In the experiment, the main channel through which competition improved market efficiency was increased patient consultations through low pricing, as opposed to the theory, which stated that competition would lead to physicians' honest treatment of patients through fair prices.

**Discussion:**

We discovered that the divergence between the theory and the experiment stemmed from the theory's reliance on the assumption that humans are rational and self-interested, which means that they are not as price-sensitive as predicted by theory.

## 1. Introduction

Supplier-induced demand (SID) is an important topic in health economics, which means that physicians can profit from information asymmetry by providing treatments against the best interests of the patient (1). SID poses a global challenge to the medical system, creates an imbalance between medical needs and deployable resources, drives up patient expenditures, and increases the probability of extreme medical expenditures (2). However, most empirical studies of SID are based on highly aggregated data, such as administrative data from hospitals (3); therefore, the empirical evidence is typically indirect. Without controlling for physician-patient interactions, it cannot be concluded from indirect evidence that such inappropriate or inefficient provision of healthcare services was truly due to the supply-side behaviors of physicians (4). This drawback also hampers the investigation of the relationship between competition and SID. Although it is well documented that hospital competition affects the quality and price of treatment, less is known about how competition affects SID. In addition, the literature shows both positive (5) and negative results (6) regarding the connection between competition and SID. Given the significant role of the market in the provision of healthcare, understanding the effect of competition on SID has substantial academic and practical value.

Research on credence goods may shed light on SID. Credence goods are services or goods for which sellers know more about the needs of consumers than themselves (7). Since consumers lack knowledge of their needs, the seller of a credence good is both an expert in diagnosing the needs of consumers and a provider of the goods or services, so the seller could use his or her information advantage to deceive consumers and gain more profits. Medical services are considered typical credence goods because patients are frequently unable to determine their illnesses and the appropriate treatment, and patients' demand for medical services depends on the diagnosis of physicians. The literature on credence goods indicates that physicians might exploit their information advantages to overtreat or undertreat patients, which is similar to the problem of SID. De Jaegher and Jegers (8) stated that the problems of credence goods and SID are identical. Compared with the literature on SID, the literature on credence goods emphasizes the asymmetric information relationship between two parties and pays more attention to the influence of institutions and market structure on physicians' behavior and equilibrium, such as the separation of diagnosis and treatment, insurance, liability and verifiability, competition, reputation, and second opinions [see (9), for a survey]. Research on credence goods makes it possible to understand how changes in institutions and market structure impact the use of information advantages and deceitful behaviors by physicians, and are also essential for comprehending the SID phenomenon. In contrast, recent updates to the credence good methodology also aid in addressing the dearth of empirical studies on SID. Darby and Karni proposed the concept of credence goods in 1973, but empirical evidence has long been lacking due to the difficulties in identifying relevant deceptive behaviors in reality (10). In the decade since the introduction of behavioral experiment methodologies by Dulleck et al. (11), a number of experimental investigations have been conducted to test these key ideas, effectively advancing the study of credence goods.

In this study, we used the framework of credence goods to analyze the impact of market competition on SID and provide evidence from behavioral experiments. In the framework of credence goods, physicians overtreat or undertreat patients to maximize their profits, and we characterize SID in terms of these two deceptive behaviors. We also introduce the heterogeneity of physician treatment costs. Previous studies assumed that both physicians and consumers are homogeneous, which ignores the role of competition in revealing information on participant types and in promoting market exit mechanisms, which may lead to underestimating the beneficial effect of competition. We assumed that there are two types of physicians' treatment costs and that patients cannot directly observe physician types. Theoretical analysis suggests that there is no honest equilibrium in a monopolistic market, while in a competitive market, physicians will reveal their cost types due to price competition, thereby generating an honest equilibrium. The experimental results indicated that competition increased the cure rate for patients by reducing prices. However, contrary to the theoretical prediction, competition increased the deceptive behavior of physicians rather than causing them to treat their patients honestly. Further analysis revealed that the reason for the inaccuracy of the theoretical prediction was that the subjects did not conform to the assumptions of homo economicus and rationality,^1^ and the subjects' low price sensitivity was responsible for the deviation of their behavior from the theoretical expectation.

This study contributes to two strands of literature. The first is the investigation into the connection between market competition and SID. Such studies frequently capture the degree of market competition based on the density of physicians (5, 6) and estimate the level of SID through variations in the income of physicians (13, 14). The findings of these studies are not entirely consistent. For example, Xirasagar and Lin (5) used data from Taiwan and found that, as the number of physicians in a given medical specialty expands, there is more quality competition among physicians, which decreases SID. However, Ikegami et al. (6) used data from MRI scans in Japan and discovered that, when the number of local hospitals that own MRI machines increases, hospitals increase the frequency of MRI scans to recover equipment costs, which also increases SID. It is difficult to compare conclusions from different studies because the context and the physician–patient interaction, including patient demand effects, communication, and the actual asymmetry of information, cannot be controlled. This study employed an experimental methodology that controlled for context and enabled direct and accurate identification of SID so that we could observe the impact of competition on SID. The other strand of literature is that on credence goods. Early studies, constrained by the difficulties in obtaining empirical evidence, were limited to theoretical analysis. Dulleck and Kerschbamer (7) summarized the relevant studies. In theory, competition would not help lessen the problem of deception because the problem of credence goods lies in the information asymmetry between sellers and consumers, which competition does not affect. Consequently, equilibrium in a competitive setting is rarely addressed in theoretical research. In recent years, the adoption of experimental methodologies has supplied vital empirical evidence and bolstered the promotion of the field. Only Dulleck et al. (11) and Mimra et al. (15) examined the role of competition through experiments and both found that price competition lowers the market price but leads to an increase in fraud by sellers. Dulleck et al. (11) and Mimra et al. (15) assumed that all experts and consumers are homogeneous. However, the assumption of homogeneity may result in an underestimation of the importance of competition because, in reality, experts are frequently distinct from one another, and competition could also contribute to the survival of the fittest. Hilger (16) explored a market with heterogeneous expert costs in which the costs are invisible to consumers. This study continues the discussion and analysis based on the assumptions of Hilger, with two differences. First, Hilger (16) obtained a weak perfect Bayesian equilibrium by carefully constructing the off-equilibrium beliefs of consumers. However, such beliefs rarely arise in the real world; hence, such a setup was abandoned in this research. Second, Hilger (16) primarily discussed the equilibrium in a monopolistic market, whereas we also analyzed the equilibrium in a competitive market.

## 2. Methods

We first constructed a theoretical model of credence goods and characterized the equilibrium. Then we designed experiments to verify the theoretical predictions.

### 2.1. Theoretical framework

A game was constructed as follows. There were two roles in the game, namely, patient and physician. Patients may suffer from minor disease *L* or severe disease *H*. The prior probability of having a severe disease was 0 < h < = 0.5. The patient could not determine the type of disease on his or her own, but the physician could do so at no cost. Moreover, the physician had two treatments to cure patients: *Treatment L* and *Treatment H*. The former could only heal disease *L*, while *Treatment H* could cure both disease *L* and disease *H*. If the disease was cured, the patient would receive benefit *v*; otherwise, he or she would receive benefit 0. We followed Hilger (16) and assumed that the cost function of physicians was unobservable to patients. There were two types of physicians, *S* and *B*, with the only difference being the treatment cost. We denoted the cost set of physicians as $C_{i}=\{C_{i},C_{i}\}$, where *C*_*i*_ is the cost of *Treatment L*, and $C_{i}$ is the cost of *Treatment H*. Each type of physician had a relative cost advantage on one treatment, whose cost set satisfied $C_{B}\leq C_{S}<C_{S}\leq C_{B}$.^2^ The physician set the price for each treatment independently, and the price should not be less than the cost. Patients could not identify the types of physicians and were simply aware that the proportion of physician *B* was *r* ∈ (0, 1). The setting of physician cost heterogeneity was realistic, and patients being unable to distinguish the types of physicians further exacerbated their information disadvantage.^3^ All aforementioned information was common knowledge to both the physician and the patient.

The action of the physician was to first set the prices of the two treatments and then choose one of the following actions: honest treatment, which was when patients received the appropriate treatment, namely, when *Treatment L* was applied to the disease *L* and *Treatment H* was applied to disease *H*; overtreatment, when *Treatment H* was applied to disease *L*; or undertreatment, when *Treatment L* was applied to disease *H*, which left the patient uncured.^4^ The actions of patients were to visit a physician and either receive treatment or refuse it. To simplify the analysis, we adopted the commitment assumption: if the patient visited a physician, he or she must accept treatment from the physician.

**Figure 1:**
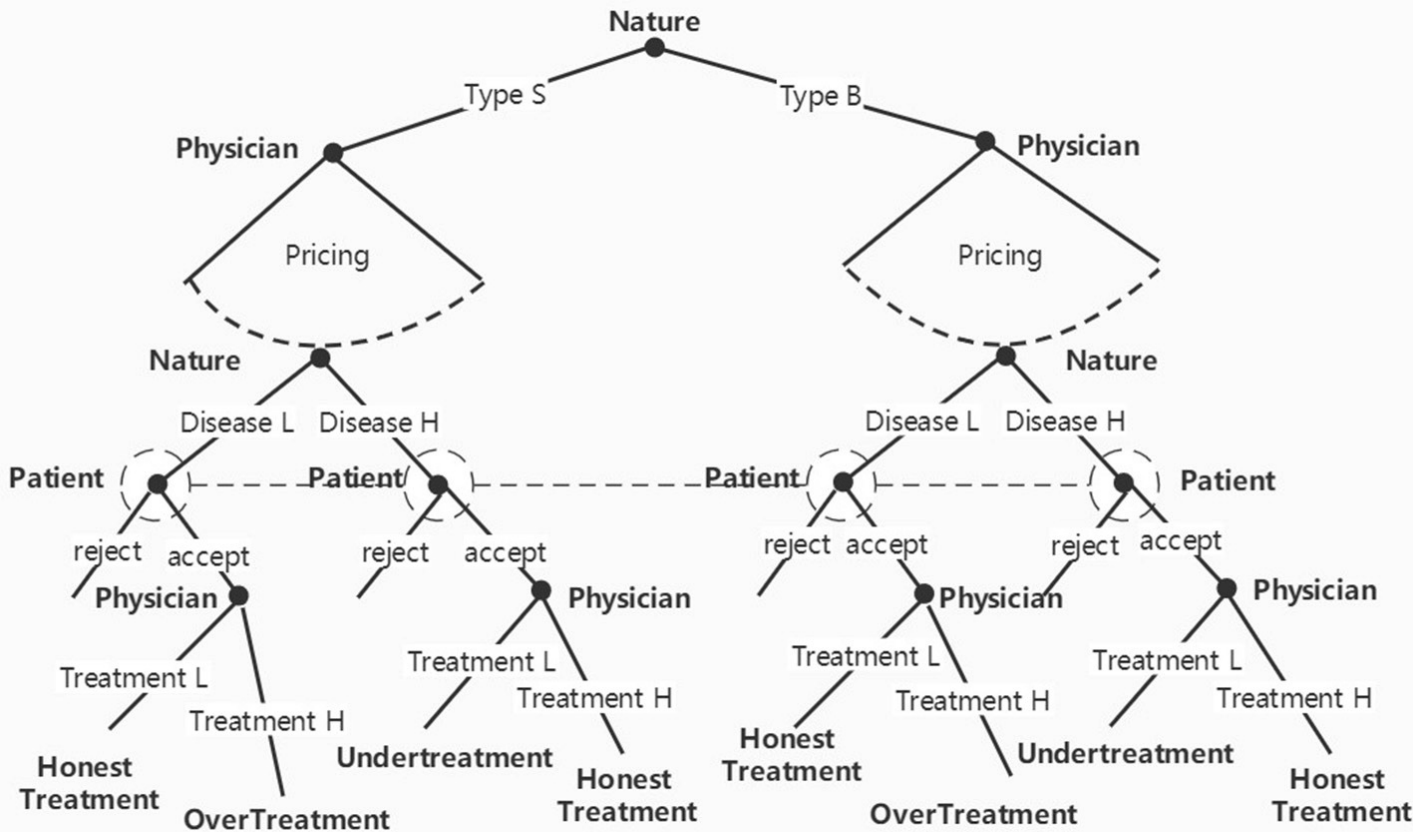
Game tree in a monopolistic market.

The game process was as follows: (1) Nature decided the type of physician and patients, which means that the types would be randomly assigned by the experiment program. (2) The physician set their prices according to his or her costs. (3) In the monopolistic market, the patient observed the price and decided whether to accept it or not; in the competitive market, the patient could observe the prices of different physicians and then decide whether and whose offer to accept. (4) If the patient chose to accept, the physician would diagnose the patient and provide the corresponding treatment; otherwise, the physician would skip to the next step. (5) The game ended, and the profit was settled. Figure 1 shows the game tree in a monopolistic market.

Ultimately, the physician obtained his or her profit from treatment, π_*Doctor*_ = *P*_*i*_ − *C*_*i*_. If no patient accepted his or her offer, the profit for the physician was 0. The payoff for the patient was the health benefits for the patient minus the treatment price, π_*Patient*_ = *v* − *P*_*i*_. If the patient received undertreatment, then π_*Patient*_ = − *P*_*H*_. If the patient declined the offer, then π_*Patient*_ = 0.

### 2.2. Equilibrium and hypotheses

**Figure 2:**
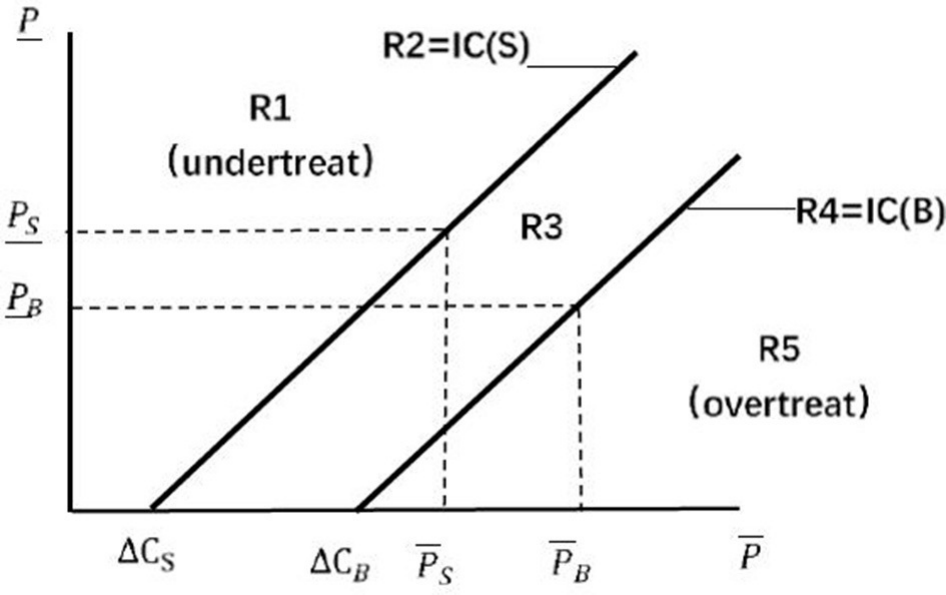
Price space (16).

According to Dulleck and Kerschbamer (7) and Hilger (16), physicians always choose the most profitable treatment. Thus, the treatment option of physicians is determined by their pricing decision. In a credence good market, physicians exploit their information advantages to overtreat or undertreat patients in pursuit of their own interests, and this deceptive behavior is the cause of SID in the medical market. In this study, we measured the SID by observing overtreatment and undertreatment by physicians. The three types of markup pricing were as follows. If the markup satisfied $\underline{P_{i}}-\underline{C_{i}}>{\bar{P}}_{i}-{\bar{C}}_{i}$, undertreating was more profitable for physicians, so we referred to this markup as the undertreat markup. Similarly, overtreat markup satisfied $\underline{P_{i}}-\underline{C_{i}}<{\bar{P}}_{i}-{\bar{C}}_{i}$, and equal markup satisfied $\underline{P_{i}}-\underline{C_{i}}={\bar{P}}_{i}-{\bar{C}}_{i}$. The analysis was conducted on the price space $\left(\underline{P_{i}},\bar{P_{i}}\right)$, which is shown in Figure 2. In Figure 2, the equal markups of the two types of physicians form two incentive compatibility (IC) lines. All price vectors on the IC (S) line are equal markups for physician *S*, any point northwest of IC (S) is the undertreat markup for physician *S*, and any point southeast of the line is the overtreat markup for physician *S*. Similarly, the IC (B) line is the IC line for physician *B*. The two IC lines divide the entire price space into five regions labeled R1–R5.

We discussed the equilibrium in monopolistic and competitive markets. In a monopolistic market, Dulleck and Kerschbamer (7) proved that an honest equilibrium exists when physicians are homogeneous, as patients would only accept equal markups from physicians, causing physicians to treat honestly. When the patient cannot observe the costs of physicians, the equilibrium is invalid, as the patient is unable to determine if the price vector of physicians represents an equal markup.

**Proposition 1: Honest equilibrium cannot exist in a monopolistic market. Neither exogenous pricing nor free pricing can achieve an honest equilibrium. However, there is a certain pricing rule: the physician will not price in region R3; if** **v <ΔC**_**S**_**/h, then the physician will not set the price in region R5; and if** **v>ΔC**_**B**_**/h, then the physician will not set the price in region R1**.

The proof can be found in the Appendix. Intuitively, we could not find a single, equal markup for physicians with different costs. Even if we set different equal markups for each type of physician, they could also increase their income by imitating the equal markup of another type. This result explained why price regulation in the medical market fails. Free pricing cannot promote honest treatment in a monopolistic market due to the inability of the price to reveal physician type, and free pricing merely increases the potential profit of physicians without limiting their deceptive behavior.

The key to eliminating deceptive behavior is to reveal the types of physicians involved. A viable alternative is employing market competition to force physicians to lower their prices until they reveal their costs. It is noticeable when there are multiple physicians of the same cost in the market, which will lead to Bertrand competition among them. Therefore, without loss of generality, we assume that there is only one physician *B* and one physician *S* in the market as, in the real world, physicians usually have their advantages, and those who do not will be driven out of the market by competition.

**Proposition 2: In a competitive market with only one physician S and one physician B, if the probability of disease ***H*** is sufficiently high ($h>\frac{{\bar{C}}_{B}-{\bar{C}}_{S}}{2(v+{\underline{C}}_{S}-{\bar{C}}_{S})}$), both types of physicians will offer an equal markup, and the patient will be able to receive honest treatment**.^5^

Refer to the proof in the Appendix for details. As the treatments provided by the two types of physicians are identical, they are competing to provide homogeneous services, which is similar to a Bertrand problem. As patients know neither their own needs nor the types of physicians, the key to the competition lies in the identification of the types of physicians. Before the price reaches the critical point $\left({\underline{C}}_{S},{\bar{C}}_{B}\right)$, price vectors cannot reveal the types of physicians. When price vectors are lower than the critical point $\left({\underline{C}}_{S},{\bar{C}}_{B}\right)$, patients can identify types of physicians by their price vectors, and then, the honest equilibrium shown by Dulleck and Kerschbamer (7) will be achieved. At the critical point, lowering prices is the dominant strategy for any type of physician. Therefore, when the two physicians in the market reach the critical point, the price vector will decrease along the IC line until one of the physicians can no longer reduce his or her price vector. Then, the patient will receive an honest diagnosis and treatment. Proposition 2 shows that competition can not only reduce the price vectors but also induce physicians to treat honestly under the premise that the competition must be intense enough to bring the price vector below the critical point $\left({\underline{C}}_{S},{\bar{C}}_{B}\right)$ for revealing types of physicians.

### 2.3. Experimental design

Based on the aforementioned theoretical model, we designed an experiment to test two hypotheses: competition can reveal types of physicians, and revealing these types can successfully encourage honest treatment. Under this objective, the experiment compared the difference between a monopolistic market and a freely priced, competitive market. We designed four experimental treatments to compare the results in different markets, as shown in Table 1. The baseline was the *Mon-Unobs* treatment, where physicians and patients were randomly matched and the patients could not distinguish the types of physicians. Moreover, we wanted to confirm the effect of the inability of patients to observe the types of the physician, so we designed the *Mon-Obs* treatment, which made the physician type visible to the patient. According to Proposition 2, we designed the *Comp* treatment: we randomly matched two patients to one physician *S* and one physician *B* in a group, and each patient could freely choose one of the physicians.^6^ In the *Comp* treatment, physicians could treat more than one patient in the group and might face situations where no patients came to them, thus they had to try to attract patients. As highlighted in Proposition 2, the basis of the honest equilibrium was that the intensity of competition forces physicians to reduce their price vectors below the critical point. However, according to the results of relevant experimental studies, we suspect that the unwillingness of subjects to lower the price may cause the competition mechanism to fail.^7^ Therefore, we constructed the *Comp-Intensive* treatment based on the *Comp* treatment, which multiplied the benefit of a physician by 10 to avoid insufficient price competition caused by the sense of fairness or the target income. Since the benefit to a physician in the *Comp-Intensive* treatment was increased in multiples, the physician may reduce the price vector below the critical point, even when considering their fairness preference.^8^ Table 1 summarizes the settings of each treatment:

**Table 1:** Treatment settings.

**Treatments**	**Visibility of physician type**	**Grouping**	**Matching method**	**Physician income multiples**
*Mon-Unobs* treatment	No	1 physician and 1 patient each	Random matching	1
*Mon-Obs* treatment	Yes	1 physician and 1 patient each	Random matching	1
*Comp* treatment	No	1 physician S, 1 physician B, and 2 patients	Random grouping, free choice within the group	1
*Comp-Intensive* treatment	No	1 physician S, 1 physician B, and 2 patients	Random grouping, free choice within the group	10

According to the theoretical model, we set the parameters as follows: the prior probability of disease *H* was *h* = 0.4. The cost of physician *S* to provide *Treatment L* and *Treatment H* was (10, 34), and the cost of physician *B* was (6, 40). The probability of receiving physician *S* was *r* = 50%. If the disease was cured, the patient could receive a health benefit *v*; otherwise, it was 0. We considered three possible values of *v*, which were 50, 70, and 90, corresponding to the three ranges of *v* in Proposition 1, with a probability of one-third for each.

Each experimental treatment consisted of 24 periods, and the experimental procedure was the same as the game sequence depicted in Figure 1. Before the session started, all subjects were randomly assigned their roles, physician or patient, which remained unchanged throughout the session. At the beginning of each period, physicians and patients were randomly matched into a group. Moreover, the types of illnesses of patients and types of physicians were randomly determined by the program according to the parameters *h* and *r*.^9^ Then, the physicians observed their types during this period and subsequently priced their treatments. After physicians offered their prices, patients checked the prices and decided whether to take the treatments. In the *Comp* or *Comp-Intensive* treatment, patients could observe the two sets of prices from two physicians in the same group, and they could choose one of them or neither. After the decisions of patients were made, the physician learnt of the illnesses of patients, then provided one of the treatments to them and charged them according to the price they offered. After the decisions of both parties were completed, the payoffs were settled and presented to the subjects, and the experiment then proceeded to the next period.

### 2.4. Experimental procedure and samples

We conducted the experiments at the Zijingang campus of Zhejiang University, and all experimental sessions were programmed using z-Tree (21). Each experimental treatment lasted two sessions, and 24 subjects participated in each session, for a total of 192 subjects from various majors. At the start of each session, we explained the experimental instructions to the subjects and had them complete tests to ensure that they understood the rules. Each experiment lasted for ~100 min, and the subjects were paid an average of RMB¥ 57.37, which was above the local average hourly wage.

## 3. Results

To test the aforementioned propositions, we focused on two strands of variables, namely, patient outcome and physician action. First, the patient outcome variables included the patient cure rate, the rejection rate, the overtreatment rate, and the undertreatment rate. The second category of variables focused on the pricing and treatment actions of physicians, including the proportion of physicians who set prices as equal markups, overtreat markups, and undertreat markups, as well as their execution of honest treatment, overtreatment, or undertreatment.

### 3.1. Sample characteristics

Table 2 shows the summary and balance check of the demographics of the subjects and other control variables. Most of the subjects in each treatment were undergraduates, and more than half of the subjects were aged 20–22, with a high proportion of science and business majors. The results of the Kruskal–Wallis test showed that there was no significant difference in the distribution of demographics of subjects among the treatments.

**Table 2:** Balance checks.

**Characteristics**	**Subjects (*****n*** = **196)**				**Kruskal-Wallis test**

	**Mon-Unobs**	**Mon-Obs**	**Comp**	**Comp-Inten**	
**Age**, ***n*** **(%)**					
<20	12 (25%)	7 (14.58%)	8 (16.67%)	7 (14.58%)	$\chi_{d.f.3}^{2}=0.975\;$
20–22	25 (52.08%)	32 (66.67%)	26 (54.17%)	29 (60.42%)	*p* = 0.807
23–25	8 (16.67%)	7 (14.58%)	12 (25%)	11 (22.92%)	
>25	3 (6.25%)	2 (4.17%)	2 (4.17%)	1 (2.08%)	
**Gender**, ***n*** **(%)**					
Men	23 (47.92%)	20 (41.67%)	22 (45.83%)	21 (43.75%)	$\chi_{d.f.3}^{2}=0.311\;$
Women	25 (52.08%)	28 (58.33%)	26 (54.17%)	27 (56.25%)	*p* = 0.958
**Major**, ***n*** **(%)**					
Medicine	9 (18.75%)	8 (16.67%)	6 (12.5%)	5 (10.42%)	$\chi_{d.f.3}^{2}=1.170\;$
Economics/business	11 (22.92%)	10 (20.83%)	8 (16.67%)	11 (22.92%)	*p* = 0.760
Science/engineering	20 (41.67%)	22 (45.83%)	26 (54.17%)	23 (47.92%)	
Philosophy/social sciences	8 (16.67%)	7 (14.58%)	7 (14.58%)	9 (18.75%)	
Other	0 (0%)	1 (2.08%)	1 (2.08%)	0 (0%)	
**Education background**, ***n*** **(%)**					
Undergraduate	35 (72.92%)	40 (83.33%)	32 (66.67%)	35 (72.92%)	$\chi_{d.f.3}^{2}=4.139\;$
Master student	8 (16.67%)	6 (12.5%)	13 (27.08%)	10 (20.83%)	*p* = 0.247
Doctoral student	5 (10.42%)	2 (4.17%)	3 (6.25%)	3 (6.25%)	
**Annual household income**, ***n*** (**%)**					
<10 k RMB	1 (2.08%)	3 (6.25%)	2 (4.17%)	1 (2.08%)	$\chi_{d.f.3}^{2}=0.960\;$
10–50 k RMB	9 (18.75%)	10 (20.83%)	14 (58.33%)	9 (18.75%)	*p* = 0.811
50–100 k RMB	21 (43.75%)	14 (58.33%)	11 (22.92%)	17 (35.42%)	
100–200 k RMB	9 (18.75%)	15 (31.25%)	14 (58.33%)	10 (20.83%)	
>200 k RMB	8 (16.67%)	6 (12.5%)	7 (14.58%)	11 (22.92%)	

### 3.2. Summary of patient outcomes

Table 3 shows the descriptive statistics of the outcomes of the patients. Compared with *Mon-Unobs* treatment, both the visibility of physician types and competition could significantly increase the cure rate of patients. The results of the Mann–Whitney U-tests indicated that the differences in the cure rate between the *Mon-Unobs* treatment and the other three treatments were statistically significant at the 1% level. The details are shown in the Appendix. In addition to the cure rate, there were also major disparities in the distribution of the treatment. In comparison to the *Mon-Unobs* treatment, the rejection rates of patients in the other three treatments were all dramatically lower, the rate of being honestly treated and that of being overtreated increased, and the rate of being undertreated decreased significantly. The difference between the competitive and monopolistic environments was more apparent: in the *Comp* treatment and the *Comp-Intensive* treatment, the rejection rate was lower than that in the two monopolistic treatments, especially in the *Comp-Intensive* treatment, in which more than 99% of the patients accepted the physician's offer. The rate of overtreatment rose sharply to more than 20% in the two competitive treatments. Overtreatment became the dominant form of deception by physicians, while in the two monopolistic settings, undertreatment was the main form of deception.

**Table 3:** Descriptive statistics of patient outcomes.

	***Mon-Unobs***	***Mon-Obs***	***Comp***	***Comp-Intensive***
Cure	54.69%	65.28%	83.16%	85.76%
Rejection	28.30%	23.78%	5.73%	0.86%
Honest treatment	49.83%	59.03%	58.33%	54.17%
Overtreatment	4.86%	6.25%	24.83%	31.60%
Undertreatment	17.01%	10.94%	11.11%	13.37%

**Conclusion 1: Compared with the** ***Mon-Unobs*** **treatment, the visibility of physician type and competition both reduced the rejection rate of patients and the rate of being undertreated and increased their rate of being overtreated, which meant that more patients were cured. The** ***Comp*** **treatment raised the patient cure rate to a greater extent than the** ***Mon-Obs*** **treatment**.

### 3.3. Physician pricing and treatment

**Figure 3:**
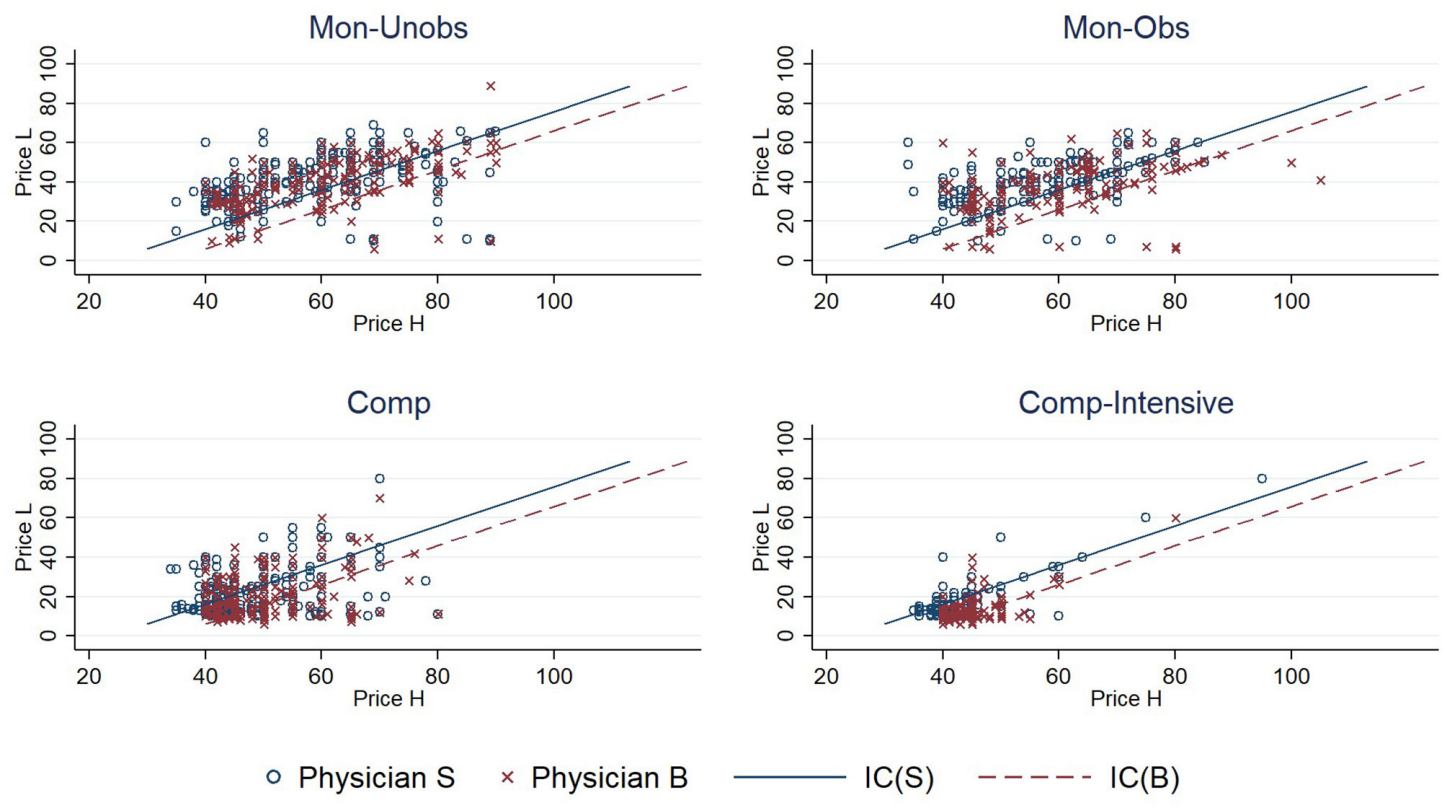
Bids of physicians in each treatment.

According to our theory, physician pricing was the key to the game. We visualized the price vectors in the experiment and compared them with theoretical predictions, as shown in Figure 3. The vertical axis represents the price of *Treatment L*, the horizontal axis is the price of *Treatment H*, the solid line represents the incentive compatibility line of physician S (IC(S)), and the dashed line is IC(B). Each point in the figure reflects one bid set by a physician.

The pricing in the experiment partially confirmed the theoretical prediction, but there was also a significant difference between them. In the *Mon-Unobs* treatment, the pricing distributions of the two types of physicians were very similar, which was consistent with the distribution in the pooling equilibrium. However, in the theoretical analysis, bidding in region R3 (the region between the two lines) was a dominant strategy. In the *Mon-Obs* treatment, prices were more concentrated than in the *Mon-Unobs* treatment, but it appeared that they were not limited to the two IC lines as predicted. Compared with the *Mon-Unobs* treatment, the proportion of biding in regions R2 and R4 increased from 6.25 to 10.16% in the *Mon-Obs* treatment. Although the direction of change was as expected, it was far from reaching the separating equilibrium of equal markups in theory. The pricing in the two competitive treatments was significantly different from that in monopolistic treatments: first, the price vectors in the competitive treatments were much lower, and second, the price vectors were concentrated in region R3. These two phenomena were more evident in the *Comp-Intensive* treatment. The points were all concentrated in the northwest corner, and 70% were in region R3. The theory also predicted that, in a competitive environment, physicians would lower their prices below the critical point (10, 40) and adopt equal markup, which was also partially supported in the *Mon-Unobs* treatment: only 1.04% of the pricing was below the critical point, compared to 6.94% in the *Comp* treatment and 28.47% in the *Comp-Intensive* treatment. Moreover, none of these pricings were equal markups. In two competitive markets, only 22.5 and 28.66% were equal markups if the price was below the critical point.

Table 4 lists the types of pricing and treatment, and it is clear that there was some correlation between them. Although only ~20% of prices were equal markup, physicians still provided over 50% honest treatment in most sessions. This propensity for honesty was also documented in the literature (11, 15, 17). Overtreat (undertreat) pricing was closely related to the overtreatment (undertreatment) behavior of physicians. Competition differentiated the pricing strategies of the two types of physicians. Physician *S* set more overtreat markups based on his or her cost advantage, while physician *B* set more undertreat markups. Affected by the pricing strategies, physicians altered their tactics of deception: physician *S* deceived patients more through overtreatment, and physician *B* deceived patients more through undertreatment. In addition, Proposition 1 contended that the health benefits of patients (*v*) would influence the pricing and treatment of physicians in *Mon-Unobs* treatment, but no significant change was observed in the experimental results. The results and analysis are shown in the Appendix.

**Table 4:** Pricing and treatment.

		***Mon-Unobs***		***Mon-Obs***		***Comp***		***Comp-Intensive***	
		***S***	***B***	***S***	***B***	***S***	***B***	***S***	***B***
Pricing type	Fair	10.76%	6.25%	20.83%	13.89%	10.07%	10.07%	17.71%	12.50%
	Overtreat	15.63%	7.29%	12.15%	5.90%	63.89%	20.49%	73.61%	14.58%
	Undertreat	73.61%	86.46%	67.01%	80.21%	26.04%	69.44%	8.68%	72.92%
Treatment	Honest	69.05%	69.95%	80.26%	74.41%	57.14%	67.19%	47.44%	63.32%
	Overtreat	8.57%	4.93%	9.65%	6.64%	35.89%	15.63%	48.40%	11.97%
	Undertreat	22.38%	25.12%	10.09%	18.96%	6.97%	17.19%	4.17%	24.71%

We used regression to analyze the impact of competition on the behavior of physicians and constructed a panel probit model as follows:


$$\begin{array}{l} y_{it}=\alpha +\beta_{1}Obs+\beta_{2}Comp+\beta_{3}Intense+\phi V_{i}+\eta_{t}+\varepsilon_{it}, \end{array}$$

Where *y*_*ip*_ is a dummy variable describing the treatment behavior of physician *i* in period *t*; *Obs*, *Comp*, and *Intense* are dummy variables that describe the visibility of physician type, market competitiveness, and competitive intensity; *V*_*i*_ is the health benefits of the patients; η_*t*_ characterizes the fixed effect of each period in the experiment; and ε_*it*_ is the error term. The data sample in the regression was the treatment behavior of physicians for all experimental treatments. If a physician had no patients receiving his or her treatment in a period, no record was generated by the physician in that period; if a physician in the *Comp* or *Comp-Intensive* treatment provided medical treatment to both patients in a period, two records were generated.

Table 5 presents the results. Row 1 shows that the visibility of types of physicians could help increase honest treatment by physicians and reduce their undertreatment, but the effect on overtreatment was not significant. Competition reduced honest treatment and undertreatment by physicians, and it significantly increased the overtreatment by physicians, which was consistent with the results in Table 4. The intensity of competition had a negative impact on the behavior of physicians, reducing their honest treatment and significantly increasing their overtreatment.

**Table 5:** Regressions of honest treatment, overtreatment, and undertreatment.

	**(1) Honest treatment**	**(2) Overtreatment**	**(3) Undertreatment**
*Obs*	0.257^***^ (0.089)	0.178 (0.173)	−0.390^***^ (0.104)
*Comp*	−0.204^**^(0.091)	0.953^***^ (0.159)	−0.510^***^ (0.088)
*Intense*	−0.196^**^ (0.083)	0.192^**^ (0.096)	0.081 (0.093)
Health benefit *v*	Yes	Yes	Yes
Period	Yes	Yes	Yes
Demography	Yes	Yes	Yes
Constant	0.727^***^ (0.197)	−2.082^***^ (0.229)	−0.691^***^ (0.227)
Number of observations	1,966	1,966	1,966

Robust standard errors in parentheses. ^***^p < 0.01 and ^**^p < 0.05.

**Conclusion 2: There was a correlation between the pricing by physicians and treatment: more overtreat (undertreat) markups led to more overtreatment (undertreatment). Equal markups occurred more in the** ***Mon-Obs*** **treatment than in the** ***Mon-Unobs*** **treatment, which induced honest treatment; in the** ***Comp*** **and** ***Comp-Intensive*** **treatments, the prices dropped significantly, and the share of equal markups rose marginally, but the honest treatment decreased**.

### 3.4. The role of competition

The results showed that deception by physicians increased in a competitive market. Although more patients were treated honestly in competitive treatments (see Table 3), the mechanism by which competition improved market outcomes was to attract more patients into the market rather than to induce honesty, where physicians set equal markups and offered honest treatment. It is clear in Table 4 that, in the *Mon-Obs, Comp*, and *Comp-Intensive* treatments, honest treatment rates were 69.5, 61.88, and 54.64%, respectively. This contradicted the theoretical prediction, and we attempted to explain why this was the case.

In our theory, there were two crucial aspects of the mechanism underlying competition: the first is that competition drives price reduction, revealing physician type, and the second is that the observability of physician type leads to equal markups and honest treatment. We checked the former first. In the *Mon-Unobs, Comp*, and *Comp-Intensive* treatments, we surveyed the beliefs of patients regarding the type of physician after their decisions about whether to accept the offer from a physician.^10^ Table 6 shows that only patients in the *Comp-Intensive* treatment could accurately identify the type of physician, and the proportion of correct guesses was >50%. However, in all three treatments, the payoff for patients with correct beliefs and those with incorrect beliefs were not considerably different. In *Comp-Intensive* treatment, competition assisted patients in properly identifying the types of physicians but it could not assist patients in improving their payoffs.

**Table 6:** Comparison of beliefs of patients and their payoffs.

	***Mon-Unobs***	***Comp***	***Comp-Intensive***
Correct guess	56.08%	54.86%	75.87%
Average payoff with the correct guess	6.25 (25.70)	25.29 (25.27)	31.34 (24.86)
Average payoff with the wrong guess	6.13 (26.31)	26.02 (23.13)	30.07 (28.18)

**Figure 4:**
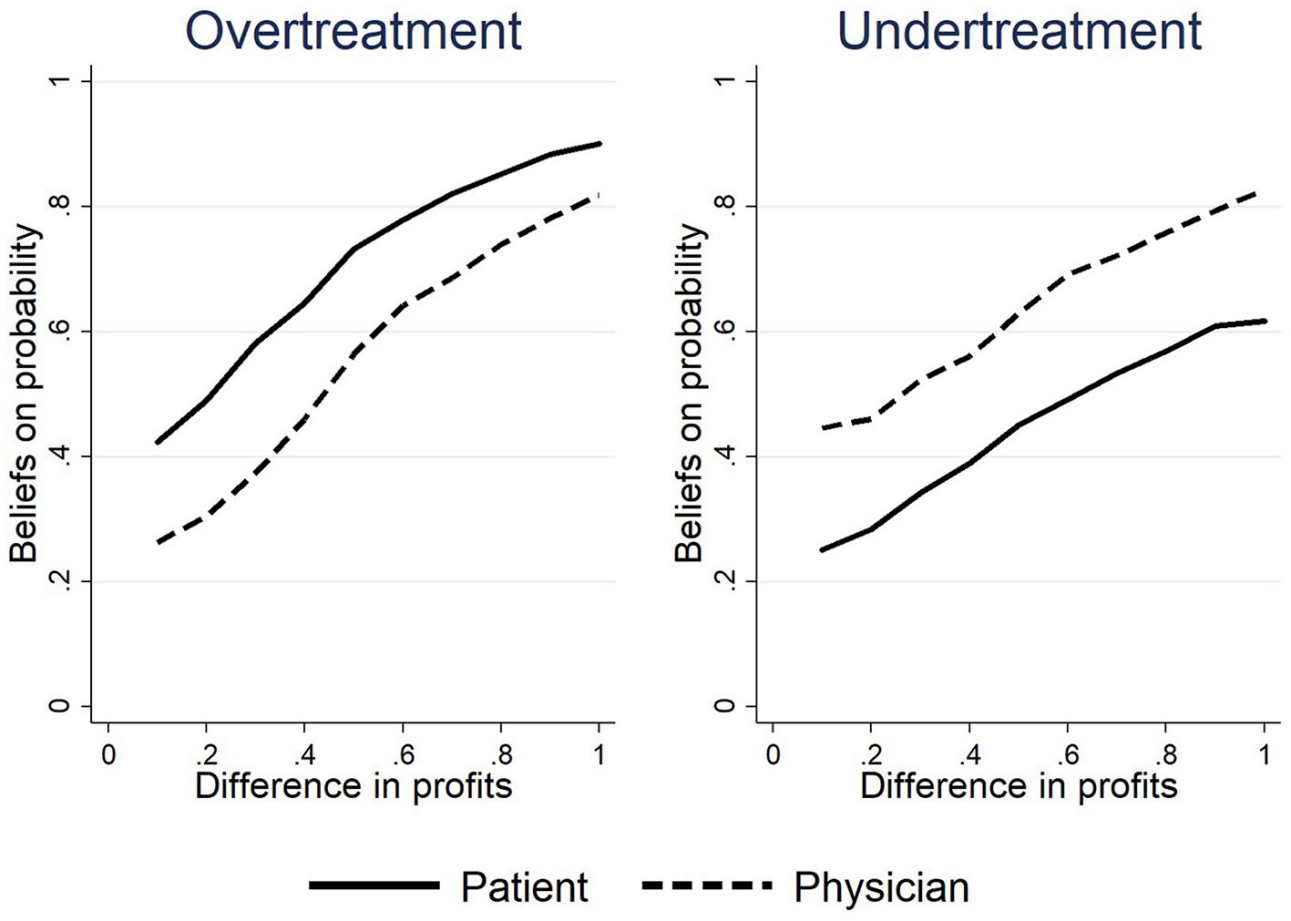
The beliefs of the correlation between markups and treatments.

Next, we turned to the second point. Table 4 shows that, after revealing their types, physicians set equal markups more frequently, consistent with the theory. Therefore, the deviation stemmed from the fact that these equal markups did not result in more honest treatments. The causality between equal markups and honest treatment derives from the homo economicus and rationality assumptions, for which we assumed that both physicians and patients believe that physicians would maximize their profits and thus provide the more profitable treatment regardless of the needs of patients. We tested this assumption through a survey in the experiments. For example, we asked physicians about the consequences of undertreat markups: “Suppose you are a physician, and your patient has a severe illness. When the profit of *Treatment L* is *N*% higher than *Treatment H*, how likely is it that you will provide *Treatment H* to the patient?” We collected the answers on a Likert scale.^11^ Figure 4 shows the results. The horizontal axis represents the difference in expected profit between the treatments, and the vertical axis represents the deception probability of respondents, which is the belief in the probability of providing profitable treatment that is inappropriate for the patient. The results revealed that both physicians and patients believed that the probability of physician deception was a continuous function that increased monotonically with the expected income gap. However, under the rationality assumption, the belief should be exactly on the line at *y* = 1, which meant that both physicians and patients did not believe that unequal markups would lead to dishonest treatment.

The real beliefs shown in Figure 4 could explain the difference between experiment and theory and reveal the actual role of competition in the experiment. The beliefs in experiments also exist in reality: physicians are trained and regulated by professional ethics, and the actual needs of patients should be the main basis for the behavior of physicians, even though economic motivation may incentivize inappropriate treatment. Patients generally trust physicians, but they are also concerned about the distortion of physician behaviors by economic motives. Such beliefs could explain the disparity between the markups and treatments by physicians: the patient trusts that there will be a considerable amount of honest treatment even at unequal markups; the physician shows his or her trustworthiness to provide honest treatment with a relatively high probability. This is a result of trust, and the results of this experiment were similar to those of the trust game (22). Furthermore, the actual beliefs of subjects could explain the deviation between the actual pricing behavior of physicians and theoretical predictions. Proposition 1 predicted that physicians would not price in region R3, and Proposition 2 predicted that physicians would reduce their prices along their IC line. This reasoning was established under the strict conditions of rational beliefs. However, due to the existence of trust, physicians may treat their patients honestly with unequal markups, and patients are willing to accept unequal markups. In this case, physicians did not need to set an equal markup, so equal markups were rare when their type was recognizable in the *Mon-Obs* and *Comp-Intensive* treatments.

**Conclusion 3: Competition may not induce honesty in physicians. Both physicians and patients believed that the probability of physician deception was a continuous function that increased monotonically with the expected income gap rather than being extremely sensitive to equal markups under the rationality assumption**.

## 4. Discussion

We will further discuss two important issues related to the validity of our results. First is the assumption regarding the competitive environment. For the sake of simplifying the analysis, this study constructed the competitive environment by analyzing only the case in which there were two physicians in the market. Although we were concerned with the shift in market equilibrium with or without competition, it would enhance the validity of our conclusion if we were to allow for more physicians in the market. On the one hand, increasing the number of physicians does not change the theoretical predictions in this study. In reality, physicians typically have their advantages, and those who do not will be driven out of the market by competition. Therefore, we must assume different cost types of physicians so that they always have an advantage in a certain treatment. To discuss the case of multiple types of physicians would require assuming more than two types of diseases and treatments, which would be too complex for theoretical analysis but would still lead to essentially the same conclusions. Wolinsky (23) analyzed the extended case of consumers with more than two types and found that the conclusions obtained in the baseline model with only two types also held in the extended model with multiple types. On the other hand, we believe that increasing the number of physicians would not affect the experimental results. Our experimental results showed that the impact of competition was first reflected in the price. As the drop in price decreased the profit margin, physicians became more likely to overtreat or undertreat to extract more profit, i.e., more SID. When there are more than two physicians in the market, more intense price competition would further reduce the profit margin, resulting in more inappropriate treatment and SID, which would be consistent with our findings.

Another topic worth considering is the disparity between the theoretical analysis and the experimental results. Both physicians and patients did not believe that profit was the primary factor influencing the treatment decisions of physicians, failing the “equal markup–honest treatment” mechanism, as shown by the analysis presented in Section Results. The external validity of this result might be challenged since the subjects we recruited were not real physicians or medical students. However, we believe that the subject identity would not strongly affect results. First, studies suggest that financial incentive has similar effects on medical students as it does on other majors, albeit to a different extent (24, 25). Second, the beliefs of the subjects shown in Figure 4 are consistent with the medical reality: although the economic incentive would distort the treatments of physicians, physicians are bound by their professional ethics, and the actual needs of patients serve as their main motivation. In addition, patients largely trust physicians, but they are also concerned about distortion in their behaviors. This conclusion responds to the theoretical controversy with behavioral evidence: some studies claim that the positive role of the competitive mechanisms should also be applicable in the medical market (26–28); others argue that the particularity of medical service information asymmetry and uncertainty causes the market structure of medical services to differ significantly from that of other environments (29), which makes it difficult for economic theory to predict the complex impact of competition on medical services (30). Our study not only corroborates the latter position but also reveals the reason why the traditional theory failed. As Hensher et al. (31) pointed out, behavioral economics can help individuals better comprehend the medical market and medical behavior.

## 5. Conclusion

In this study, we used the framework of credence goods to analyze the impact of market competition on SID and provide empirical evidence through behavioral experiments. In our theoretical analysis, there was no honest equilibrium in a monopolistic market, while in a competitive market, price competition could reveal the types of physicians and thus induce an honest equilibrium. The experimental results showed that competition increased the consultations of patients by lowering the price, which eventually resulted in more patients being cured. However, compared with the monopolistic market, competition induced more SID (overtreatment or undertreatment by physicians in our context) rather than reducing it. The results of the experiment were consistent with the findings of Sørensen and Grytten (32) and Ikegami et al. (6), and further expanded the application of their conclusions. The literature focuses on regulated prices, which leads to quality competition. We allowed physicians to freely set prices for their treatments, so physicians competed mainly on price. Combining the experimental results of this study and the literature, it can be concluded that both quality and price competition lead to more SID. This study also specifically demonstrated the connection between price cuts by physicians and SID under price competition.

The most important implication of this study is to point out that marketization is not a panacea for problems in the healthcare system. We also explained why market competition alone is insufficient to overcome SID. The “market” has become a multi-purpose toolbox in public services, and policymakers now have four decades of experience using marketization to address cost and quality problems in public-sector health services. However, the consequences of marketization are uncertain, with conflicting evidence about its effects on service cost and quality. Therefore, it is conceivable for policymakers to misinterpret the effect of marketization on the medical market. The model and experiments in this study provide a clear abstraction from reality, enabling us to directly observe how the competition works. This allows policymakers to make a reasonable assessment of marketization policy. Our results suggest that marketization may not be an appropriate solution to the SID problem, or at the very least, its theoretical foundations are questionable. Policymakers should recognize that marketization could reduce prices, encourage patients to have more physician consultations, increase the efficiency of healthcare services, and enhance the welfare of patients, but it is not guaranteed to encourage physicians to provide suitable treatments, reduce the expenses of patients, or get rid of SID. Policymakers should turn their attention away from marketization when dealing with quality problems in public-sector medical services.

## Data availability statement

The raw data supporting the conclusions of this article will be made available by the authors, without undue reservation.

## Ethics statement

The studies involving human participants were reviewed and approved by Interdisciplinary Center for Social Sciences, Zhejiang University. The Ethics Committee waived the requirement of written informed consent for participation.

## Author contributions

YC, YP, and YD contributed to conception and design of the study. YP and YD programmed the experiments. YD organized the experiment, performed the statistical analysis, and wrote the first draft of the manuscript. All authors contributed to manuscript revision, read, and approved the submitted version.

## Footnotes

^1^Homo economicus, or economic man, is the portrayal of humans as agents who are consistently rational and narrowly self-interested [see Thaler (12) for a survey].

^2^Subjects at an absolute disadvantage in the market would be eliminated from the market, so we assumed that physicians with heterogeneous costs had a relative cost advantage on one treatment. Here, we followed the assumptions of Hilger (16).

^3^It is true that the most common heterogeneity among physicians is in skill, often expressed as the probability of correct diagnosis and successful treatment. However, because uncertainty in diagnostic and treatment outcomes can interfere with judgments and observations of subjective deceptive behavior of physicians, this study needed to exclude uncertainty. In addition, heterogeneity in skill can also manifest as differences in cost, with less skilled physicians requiring more examinations and medicines to diagnose and cure patients, all of which may lead to differences in treatment costs.

^4^The verifiability assumption in credence goods was introduced here, which required that the treatment provided by the physician must be consistent with the price he or she charges. The fact that all the patients' expenditures at the healthcare facility were documented was a realistic manifestation of the verifiability assumption.

^5^When $h<\frac{{\bar{C}}_{B}-{\bar{C}}_{S}}{2(v+{\underline{C}}_{S}-{\bar{C}}_{S})}$, the result is that both physicians stay at the critical point and the honest equilibrium does not exist. However, according to numerical simulation, the critical point is very small, so the probability of the occurrence of disease H (*h*) is unlikely to fall in the region.

^6^In our experiment, the identity of subjects could not be recognized between periods to avoid the effect of reputation, and subjects were not allowed to communicate during the session to avoid the influence of cheap talk, which means communication without any commitment.

^7^In experimental studies of credence goods, subjects who played the role of monopolistic experts do not capture the full surplus of consumers as argued in theoretical predictions (11, 15, 17). It is possible that fair preferences of subjects yielded this result, and the right of consumer to reject in the credence good game was similar to that in the ultimatum game (18). Additionally, the target income hypothesis in SID studies proposes that physicians have income targets that may influence their behavior (19, 20). Therefore, we were concerned that physicians might not reduce their price vectors below the critical point in the *Comp* treatment because their returns are relatively low.

^8^It is clear that other preferences may also influence the experimental results. This study specifically designed the *Comp-Intensive* treatment only based on the preference for fairness because it may impede the price reduction as has been directly confirmed in previous experiments. Other preferences may not have such a direct effect.

^9^We set *h*=*0.4* in the experiment, which meant that 40% of patients were randomly assigned severe diseases and the other 60% minor diseases. In addition, *r*=*0.5* meant that half of the physicians were randomly assigned to type *B*, and the other half were type *S*. In *Comp* and *Comp-Intensive* treatment, there must be one type *S* and one type *B* in a group.

^10^In the *Mon-Unobs* treatment, we asked patients for their beliefs about the type of the matched physician in the current period; in the *Comp* treatment and *Comp-Intensive* treatment, where subjects explicitly knew that the types of the two physicians in group were one each of type S and type B, we asked patients for their beliefs regarding the type of the physician on the left side of the screen (the bids of the two physicians were displayed on the left and right sides of the screen). In addition, we incentivized this report. We informed the subjects that a bonus period would be drawn at the end of the experiment and that they would be rewarded with experiment points if they were correct about their belief regarding type of physician in that period. The reward was 300 experiment points, which accounted for approximately 30%−70% of the total gain of the patient.

^11^The questionnaire asked in detail about the impact of unequal markups on subjects with different degrees of profit variation, and the N% in the question was taken to range from 10% to 100%, increasing in steps of 10%.
