# Supplementary material for: How does market competition affect supplier-induced demand? An experimental study
Source: Front Public Health. 2023 Mar 8;11:1024337. doi: 10.3389/fpubh.2023.1024337 (PMC10030521; doi:10.3389/fpubh.2023.1024337)
Supplement: Supplementary file 1 [file Table_1.DOCX]

Supplementary Material

# Supplementary Omitted Proofs

## Proof of Proposition 1

1. The absence of one or more price vectors that make both types of physicians honest

Counterfactual method. Suppose there exists a price vector $(\underline{P}^{*},\bar{P}^{*})$ which make both types of physicians treat their patients honestly, then the benefits of providing treatment H or L at that price should be equal for both types of physicians, then both should satisfy$\underline{P}^{*}-\underline{C}_{S}=\bar{P}^{*}-\bar{C}_{S}$, and $\underline{P}^{*}-\underline{C}_{B}=\bar{P}^{*}-\bar{C}_{B}$ . This is not possible under our basic assumption $\underline{C}_{B}\leq\underline{C}_{S}<\bar{C}_{S}\leq\bar{C}_{B}$.

Consider multiple price vectors. In a monopoly market, physicians will set their prices at the patient's constraint level and make patient's expected benefit zero, so physicians will capture the full market welfare. If an honest separating equilibrium exists, the patient's willingness to pay is v. If Physician S treats honestly, his markup is $\underline{P}_{S}=v-h\cdot\Delta C_{S}$ , $\overline{P}_{S}=v+\left( 1-h \right)\cdot\Delta C_{S}$ , Physician S can earn a profit$\pi_{S}=v-\underline{C}_{S}-h\cdot\Delta C_{S}$, where $\Delta C_{i}=\bar{C}_{i}-\underline{C}_{i}$, denotes the cost difference between the two treatments of the physician. If Physician B treats honestly, his markup is $\underline{P}_{B}=v-h\cdot\Delta C_{B}$, $\overline{P}_{B}=v+\left( 1-h \right)\cdot\Delta C_{B}$, Physician B can earn a profit $\pi_{B}=v-\underline{C}_{B}-h\cdot\Delta C_{B}$. But the physician can also imitate the equal markup of another type of physician and cheat the patient at that price: if Physician S imitates the equal markup of Physician B, he will overtreats the patient, which makes his profit ${\pi'}_{S}=v+\left( 1-h \right)\cdot\Delta C_{B}-\overline{C}_{S}$. If Physician B imitates Physician S's equal markup, he will definitely undertreat his patients, and his profit will be ${\pi'}_{B}=v-h\cdot\Delta C_{s}-\underline{C}_{B}$.

If a separation equilibrium exists in which both physicians are honest, physicians’ expected benefit of honest treatment must be greater than that of deceptive treatment, i.e.$\pi_{S}>{\pi'}_{S}$, $\pi_{B}>{\pi'}_{B}$ must hold. Both of the preceding inequalities work out to be $\Delta C_{S}>\Delta C_{B}$, which contradicts the basic assumptions $\Delta C_{S}<\Delta C_{B}$ of this paper. Therefore, it can be shown that the separating equilibrium that makes both types of physicians honest does not exist.

1. The result of monopoly market when free pricing

The maximum profits with corresponding markups for the two types of physicians in region R1-R5 under monopoly conditions are as follow:

In region R1, both physician S and B will undertreat. The participation constraint of patients satisfies $\left( 1-h \right)v=\underline{P}$. Then we can calculate the prices and profits:$\underline{P}=\left( 1-h \right)v$, $\bar{P}<\left( 1-h \right)v+\Delta C_{S}$, $\pi_{S}=\left( 1-h \right)v-\underline{C}_{S}$, $\pi_{B}=\left( 1-h \right)v-\underline{C}_{B}$ .

In region R2, Physician S will treat honestly and Physician B will undertreat, and patient's participation constraint satisfies $\left( 1-rh \right)v=\left[ 1-h\left( 1-r \right) \right]\underline{P}+h\left( 1-r \right)\bar{P}$. Then we can calculate the prices and profits: $P_{L}=\left( 1-rh \right)v-h\left( 1-r \right)\Delta C_{S}$, $P_{H}=\left( 1-rh \right)v+[1-h\left( 1-r \right)]\Delta C_{S}$, $\pi_{S}=\left( 1-rh \right)v+rh\Delta C_{S}-h\overline{C}_{S}-\left( 1-h \right)\underline{C}_{S}$, $\pi_{B}=\left( 1-rh \right)v-h\left( 1-r \right)\Delta C_{S}-\underline{C}_{B}$.

In region R3, Physician S will overtreat and Physician B will undertreated, and patient's participation constraint satisfies $\left( 1-rh \right)v=r\underline{P}+\left( 1-r \right)\overline{P}.$ Then we can calculate the prices and profits: $\overline{P}-\Delta C_{B}<\underline{P}<\overline{P}-\Delta C_{S}$, $\pi_{S}=\overline{P}-\overline{C}_{S}$, and $\pi_{B}=\underline{P}-\underline{C}_{B}$ .

In region R4, Physician S well overtreat and Physician B will honestly treat, and the patient's participation constraint satisfies $v=r\left( 1-h \right)\underline{P}+\left( 1-r+rh \right)\overline{P}$. Then we can calculate the prices and profits: $P_{L}=v-\left[ 1-r\left( 1-h \right) \right]\Delta C_{B}$, $P_{H}=v+r\left( 1-h \right)\Delta C_{B}$, $\pi_{S}=v+r\left( 1-h \right)\Delta C_{B}-\overline{C}_{S}$, and $\pi_{B}=v-\left( 1-r \right)\left( 1-h \right)\Delta C_{B}-h\overline{C}_{B}-\left( 1-h \right)\underline{C}_{B}$.

In region R5, both Physicians S and B will overtreat, and the patient participation constraint satisfies $v=\overline{P}$. Then we can calculate the prices and profits: $P_{L}<v-\Delta_{B}$, $P_{H}=v$, $\pi_{S}=v-\overline{C}_{S}$, $\pi_{B}=v-\overline{C}_{B}$.

Here are the proofs:

a) Physicians will never bid in region R3: in region R3 Physician B will always undertreat and Physician S will always overtreat. Pricing $\underline{P}=\left( 1-h \right)v$ may lead highest profit to Physician B when he undertreats, and pricing$\bar{P}=v$ may lead highest profit to Physician S when he overtreats. As price vector ($\left( 1-h \right)v，v$) is not in region R3, thus $\underline{P}≶\left( 1-h \right)v$ and $\overline{P}≶h$ divides region R3 into four areas.

When $\underline{P}<\left( 1-h \right)v$ and $\overline{P}<h$, Physician B will deviate to region R1 and Physician S will deviate to region R5 for higher profit.

When $\underline{P}>\left( 1-h \right)v$ and $\overline{P}>h$, patient will reject all bids because the patient's expected benefit is $\left( 1-rh \right)v-r\underline{P}-\left( 1-r \right)\overline{P}<0$ .

When $\underline{P}>\left( 1-h \right)v$ and $\overline{P}<h$, Physician B will deviate to region R1 for higher profit.

When $\underline{P}<\left( 1-h \right)v$ and $\overline{P}>h$, Physician S will deviate to region R5 for higher profit.

Therefore, there is no equilibrium in region R3.

b) If there is $v<\frac{\Delta C_{s}}{h}$, then the physician will not bid in the region R5: to make the profits in region R1 for both types of physicians higher than that in region R5, it require $\pi_{S}\left( R1P \right)>\pi_{S}(R5P)$ and $\pi_{B}\left( R1P \right)>\pi_{B}(R5P)$, which yields$v<\frac{\Delta C_{S}}{h}$. That is, if satisfies $v<\frac{\Delta C_{S}}{h}$, the bids in region R1 is strictly dominant over any bid in region R5.

c) If there is $v>\frac{\Delta C_{B}}{h}$, then the physicians will not bid in the region R1: to make the profits in region R5 for both types of physicians higher than that in region R1, it requires $\pi_{S}\left( R5P \right)>\pi_{S}(R1P)$ and $\pi_{B}\left( R5P \right)>\pi_{B}(R1P)$, which yields $v>\frac{\Delta C_{B}}{h}$. That is, if satisfies $v>\frac{\Delta C_{B}}{h}$, the bids in region R5 is strictly dominant over any bid in region R1.

## Proof of Proposition 2

The two types of physicians provide exactly the same two treatments L and H. Thus, Physician S and Physician B in this market are still competing on price for homogeneous services. But the invisibility to costs of physicians make it possible for patients to not accurately identify the type of physician, making it difficult for patients to accurately calculate expected benefits. When patients are able to accurately recognize the type of physician, the competitive market is similar to the Bertrand problem. Thus, the key to the competitive market equilibrium is the ability to accurately reveal physician type.

Obviously, before the markup drops to$\left( \underline{C}_{S},\overline{C}_{B} \right)$, neither physician will offer an equal markup which satisfies $\underline{P}_{i}-\underline{C}_{i}=\overline{P}_{i}-\bar{C}_{i}$, because another physician can mimic this equal markup and the patient cannot identify the type of physician. This imitation is profitable: suppose that Physician S sets an equal markup $\left( \underline{C}_{S}+x, \overline{C}_{S}+x \right)$ which satisfies $\overline{C}_{S}+x>\overline{C}_{B}$, then the corresponding equal markup for Physician B is $\left( \underline{C}_{B}+x, \overline{C}_{B}+x \right)$, satisfying $\underline{C}_{B}+x>\underline{C}_{S}$, and Physician S and Physician B can obtain the same expected benefit $\pi_{S}=\pi_{B}=x$ under both equal markups. If Physician B imitates Physician S's markup, his expected profit will be $\bar{\pi}_{B}=\underline{C}_{S}-\underline{C}_{B}+x>\pi_{B}$, so Physician B has an incentive to imitate Physician S's equal markup. Similarly, it follows that Physician S also has an incentive to imitate physician B's equal markup. Thus the equal markups above $\left( \underline{C}_{S},\overline{C}_{B} \right)$ cannot show the type of physician, and giving an equal markup in this interval does not provide a competitive advantage to the physician.

Since physicians are not allowed to bid below their cost, when the price drops to$\left( \underline{C}_{S},\overline{C}_{B} \right)$, physicians will show their type if they continue to lower his markup, then patients can accurately recognize the physician's type. When a physician accurately displays his type, he will inevitably set an equal markup, because under which the physician will certainly treat honestly and make no welfare loss in the market, while the welfare loss caused by overtreatment or undertreatment under an unequal markup will become a disadvantage in competition for the physician. The price of a physician's disadvantageous treatment under equal markups will be higher than $\left( \underline{C}_{S},\overline{C}_{B} \right)$ point: Physician S appears $\underline{P}_{S}>\underline{C}_{S}$, Physician B appears $\bar{P}_{B}>\overline{C}_{B}$. If a physician decides to price the advantageous service below$\left( \underline{C}_{S},\overline{C}_{B} \right)$, then patients are able to accurately recognize the type of physician and calculate their expected benefits, the market becomes a Bertrand market.

$\left( \underline{C}_{S},\overline{C}_{B} \right)$is the key point. At this price point, if physicians do not continue to lower prices then both types of physicians gain through overtreatment or undertreatment; if physicians decide to lower prices then the market engages in Bertrand competition and the overall higher-cost physician's gain will be 0. Therefore the focus is on examining the price changes at $\left( \underline{C}_{S},\overline{C}_{B} \right)$ point on the price reduction choices of the two types of physicians to explore the possibility of a collusion situation where neither physician reduces prices.

**I.** The overall cost of Physician S is lower, i.e. $\left( 1-h \right)\underline{C}_{S}+h\overline{C}_{S}<\left( 1-h \right)\underline{C}_{B}+h\overline{C}_{B}$.

1. If both physicians do not continue to reduce the price and the price remains at $(\underline{C}_{S},\overline{C}_{B})$, Physician B is always undertreating and Physician S is always overtreating. The profits of the two physicians are $\pi_{B}=\underline{C}_{S}-\underline{C}_{B}$ and $\pi_{S}=\overline{C}_{B}-\overline{C}_{S}$ .
2. If both physicians choose to reduce their prices, they will mark-down along each IC lines, Physician S will win the Bertrand competition due to lower overall costs, and Physician S's expected profit is equal to the overall cost difference between Physician S and Physician B. The expected profit is $\pi_{B}=0$, and $\pi_{S}=2[\left( 1-h \right)\left( \underline{C}_{B}-\underline{C}_{S} \right)+h\left( \overline{C}_{B}-\overline{C}_{S} \right)]$.
3. If Physician S decides to reduce his price but Physician B does not, the patient is able to accurately recognize the type of physician and expects that he or she will receive earnings $\left( 1-h \right)v-\underline{C}_{S}$ from Physician B. Assume that the equal markup given by Physician S is $(\underline{C}_{S}+x, \overline{C}_{S}+x)$, then the patient's expected gain from physician S is $v-\left( 1-h \right)\left( \underline{C}_{S}+x \right)-h(\overline{C}_{S}+x)$. If Physician S makes the patient choose him, he should provide the patient a higher earning than that from Physician B, which means $v-\left( 1-h \right)\underline{C}_{S}-h\overline{C}_{S}-x^{*}=\left( 1-h \right)v-\underline{C}_{S}\Longleftrightarrow x^{*}=h(v+\underline{C}_{S}-\overline{C}_{S})$.
4. If $\overline{C}_{S}+x^{*}<\overline{C}_{B}$ , then the price vector can show the type of physicians to patients. The expected profit for Physician S is $\pi_{S}=2x=2h(v+\underline{C}_{S}-\overline{C}_{S})$, and the expected profit for Physician B $\pi_{B}=0$.
5. If $\overline{C}_{S}+x^{*}>\overline{C}_{B}$, the relative cost advantage of Physician S is greater, so Physician S needs to continue to reduce prices by $(\underline{C}_{S}+\overline{C}_{B}-\overline{C}_{S}, \overline{C}_{B}-\varepsilon)$, where $\varepsilon\to0$, the expected earning to patients at this point of choosing physician S is definitely higher than that of choosing Physician B. The expected profit to Physician S if ${\pi'}_{S}=2(\overline{C}_{B}-\overline{C}_{S})$.
6. If Physician B decides to reduce the price but physician S does not, the patient is able to accurately recognize the type of physician and expects that he or she will receive earnings $v-\overline{C}_{B}$ from Physician S. Assume that the equal markup given by Physician B is $(\underline{C}_{B}+x, \overline{C}_{B}+x)$, the patient's expected gain from Physician B is $v-\left( 1-h \right)\left( \underline{C}_{B}+x \right)-h(\overline{C}_{B}+x)$. If Physician B makes the patient choose him, he should provide the patient a higher earning than that from Physician S, which means $v-\left( 1-h \right)\underline{C}_{B}-h\overline{C}_{B}-x^{*}=v-\overline{C}_{B}\Longleftrightarrow x'=(1-h)(\overline{C}_{B}-\underline{C}_{B})$ .

Under the assumption that the overall cost of Physician S is lower, there is $\left( 1-h \right){(\underline{C}}_{S}-\underline{C}_{B})<h(\overline{C}_{B}-\overline{C}_{S})$. And because$h\in(0,0.5]$, there is$1-h>h$, we therefore obtain $\underline{C}_{S}-\underline{C}_{B}<\overline{C}_{B}-\overline{C}_{S}$. It is further known that $\overline{C}_{B}-\underline{C}_{B}>2(\underline{C}_{S}-\underline{C}_{B})$ holds, then there must be $\left( 1-h \right)\left( \overline{C}_{B}-\underline{C}_{B} \right)>\underline{C}_{S}-\underline{C}_{B}$, which means $\underline{C}_{B}+x'>\underline{C}_{S}$ must hold. Then Physician B needs to continue to reduce the price by $(\underline{C}_{S}-\varepsilon, \overline{C}_{B}+\underline{C}_{S}-\underline{C}_{B})$, where$\varepsilon\to0$. Then the patient's expected earning of choosing Physician B must be higher than that of choosing S. The expected benefit of physician B,${\pi'}_{B}=2(\underline{C}_{S}-\underline{C}_{B})$ .

Based on the analysis above, the payment matrix is obtained as follows.

| Table A1. Payment matrix when Physician S with lower overall cost | | | |
| --- | --- | --- | --- |
|  |  | Physician B | |
|  |  | Price reduction | No price reduction |
| Physician S | Price reduction | 0 | 0 |
|  |  | $2[\left( 1-h \right)\left( \underline{C}_{B}-\underline{C}_{S} \right)$  $+h\left( \overline{C}_{B}-\overline{C}_{S} \right)]$ | $2h(v+\underline{C}_{S}-\overline{C}_{S})$  or$2(\overline{C}_{B}-\overline{C}_{S})$ |
|  | No price reduction | $2(\underline{C}_{S}-\underline{C}_{B})$ | $\underline{C}_{S}-\underline{C}_{B}$ |
|  |  | 0 | $\overline{C}_{B}-\overline{C}_{S}$ |

Analyze the dominant strategy for physicians based on the payment matrix above:

1. When Physician B does not reduce his price, Physician S's dominant strategy is to reduce his price. Compare the profits of Physician S when the price is reduced or not: since $h>\frac{\overline{C}_{B}-\overline{C}_{S}}{2(v+\underline{C}_{S}-\overline{C}_{S})}$, therefore both $2h\left( v+\underline{C}_{S}-\overline{C}_{S} \right)-\left( \overline{C}_{B}-\overline{C}_{S} \right)>0$ and $2\left( \overline{C}_{B}-\overline{C}_{S} \right)-\left( \overline{C}_{B}-\overline{C}_{S} \right)=\overline{C}_{B}-\overline{C}_{S}>0$ hold, the profit of Physician S is higher with a price cut.
2. When Physician B reduces his price, Physician S's dominant strategy is to reduce his price. Obviously, there is$2\left[ \left( 1-h \right)\left( \underline{C}_{B}-\underline{C}_{S} \right)+h\left( \overline{C}_{B}-\overline{C}_{S} \right) \right]>0$, the payoff of Physician S is higher with a price cut.
3. When Physician S does not reduce his price, Physician B's dominant strategy is to reduce his price. Comparing the payoffs when physician B lowers his price with those when he does not: $2\left( \underline{C}_{S}-\underline{C}_{B} \right)-\left( \underline{C}_{S}-\underline{C}_{B} \right)=\underline{C}_{S}-\underline{C}_{B}>0$, the payoff of price reduction for Physician B is higher.
4. When Physician S reduces his price, Physician B's expected benefit is 0 regardless of whether he reduces his price or not, and it makes no difference whether Physician B reduces his price or not.

The above analysis shows that the dominant strategy for both Physician S and Physician B is to reduce prices, both types of physicians will give equal markups, and show their type. Therefore the market will continue to reduce prices along the Bertrand competitive path until it falls to the cost level of physician B, who has a higher overall cost $(\underline{C}_{B}, \overline{C}_{B})$. Patients will receive honest diagnosis and treatment.

**II.** The overall cost of physician B is lower, i.e.$\left( 1-h \right)\underline{C}_{S}+h\overline{C}_{S}>\left( 1-h \right)\underline{C}_{B}+h\overline{C}_{B}$.

1. If both physicians do not continue to reduce the price and the price remains at $(\underline{C}_{S},\overline{C}_{B})$, Physician B is always undertreating and Physician S is always overtreating. The profits of the two physicians are $\pi_{B}=\underline{C}_{S}-\underline{C}_{B}$ and$\pi_{S}=\overline{C}_{B}-\overline{C}_{S}$.
2. If both physicians choose to reduce their prices, they will mark-down along each IC lines, Physician B will win the Bertrand competition due to lower overall costs, and physician B's expected profit is equal to the overall cost difference between Physician S and Physician B. The expected profit is $\pi_{S}=0$, and $\pi_{B}=2[\left( 1-h \right)\left( \underline{C}_{S}-\underline{C}_{B} \right)+h\left( \overline{C}_{S}-\overline{C}_{B} \right)]$.
3. If Physician B decides to reduce the price but Physician S does not, the patient is able to accurately recognize the type of physician and expects that he or she will receive earnings $v-\overline{C}_{B}$ from Physician S. Assume that the equal markup offered by physician B is $(\underline{C}_{B}+x, \overline{C}_{B}+x)$ , the patient's expected gain at physician B is $v-\left( 1-h \right)\left( \underline{C}_{B}+x \right)-h(\overline{C}_{B}+x)$. If Physician B makes the patient choose him, he should provide the patient a higher earning than that from Physician S, which means $v-\left( 1-h \right)\underline{C}_{B}-h\overline{C}_{B}-x^{*}=v-\overline{C}_{B}\Longleftrightarrow x^{*}=(1-h)(\overline{C}_{B}-\underline{C}_{B})$ .
4. If $\underline{C}_{B}+x^{*}<\underline{C}_{S}$ , then the price vector can show the type of physicians to patients. The expected profit for Physician B is $\pi_{B}=2x=2(1-h)(\overline{C}_{B}-\underline{C}_{B})$, and the expected profit for Physician S is $\pi_{S}=0$.
5. If $\underline{C}_{B}+x^{*}>\underline{C}_{S}$, then Physician B needs to continue to reduce the price until pricing $(\underline{C}_{S}-\varepsilon, \overline{C}_{B}+\underline{C}_{S}-\underline{C}_{B})$, where $\varepsilon\to0$. at this bid the patient's expected earning of choosing Pysician B must be higher than that of choosing Physician S. The expected profit of Physician B is $\pi_{B}=2(\underline{C}_{S}-\underline{C}_{B})$ and the expected benefit of Physician S is $\pi_{S}=0$.
6. If Physician S decides to reduce the price but Physician B does not, the patient is able to accurately recognize the type of physician and expects that he or she will receive earnings $\left( 1-h \right)v-\underline{C}_{S}$ from Physician B. Assume that the equal markup given by Physician S is $(\underline{C}_{S}+x, \overline{C}_{S}+x)$, the patient's expected gain from Physician S is $v-\left( 1-h \right)\left( \underline{C}_{S}+x \right)-h(\overline{C}_{S}+x)$. If Physician S makes the patient choose him, he should provide the patient a higher earning than that from Physician B, which means$v-\left( 1-h \right)\underline{C}_{S}-h\overline{C}_{S}-x'=\left( 1-h \right)v-\underline{C}_{S}\Longleftrightarrow x'=h(v+\underline{C}_{S}-\overline{C}_{S})$ .
7. If $\overline{C}_{S}+x'<\overline{C}_{B}$, the price vector can show physicians’ type to patients. The expected profit of Physician S is $\pi_{S}=2x=2h(v+\underline{C}_{S}-\overline{C}_{S})$, the expected profit of Physician B is $\pi_{B}=0$.
8. If $\overline{C}_{S}+x'>\overline{C}_{B}$, as the relative cost advantage of Physician S is greater, Physician S needs to continue to reduce prices until pricing $(\underline{C}_{S}+\overline{C}_{B}-\overline{C}_{S}, \overline{C}_{B}-\varepsilon)$, where$\varepsilon\to0$. The expected earnings to patients of choosing Physician S is definitely higher than that of choosing Physician B. The expected profit to Physician S is $\pi_{S}=2(\overline{C}_{B}-\overline{C}_{S})$ and the expected benefit of Physician B is $\pi_{B}=0$.

Based on the analysis above, the payment matrix is obtained as follows.

| Table A2. Payment matrix when Physician B with lower overall cost | | | |
| --- | --- | --- | --- |
|  |  | Physician B | |
|  |  | Price reduction | No price reduction |
| Physician S | Price reduction | $2[\left( 1-h \right)\left( \overline{C}_{S}-\underline{C}_{B} \right)+h\left( \overline{C}_{S}-\overline{C}_{B} \right)]$ | 0 |
|  |  | 0 | $2h(v+\underline{C}_{S}-\overline{C}_{S})$  or $2(\overline{C}_{B}-\overline{C}_{S})$ |
|  | No price reduction | $2\left( 1-h \right)\left( \overline{C}_{B}-\underline{C}_{B} \right)$  or $2(\underline{C}_{S}-\underline{C}_{B})$ | $\underline{C}_{S}-\underline{C}_{B}$ |
|  |  | 0 | $\overline{C}_{B}-\overline{C}_{S}$ |

Analyze the dominant strategy for physicians based on the payment matrix above:

1. When Physician B does not reduce his price, the dominant strategy for Physician S is to reduce his price. Compare the profit of Physician S when price is reduced or not: since $h>\frac{\overline{C}_{B}-\overline{C}_{S}}{2(v+\underline{C}_{S}-\overline{C}_{S})}$, therefore both$2h\left( v+\underline{C}_{S}-\overline{C}_{S} \right)-\left( \overline{C}_{B}-\overline{C}_{S} \right)>0$ and $2\left( \overline{C}_{B}-\overline{C}_{S} \right)-\left( \overline{C}_{B}-\overline{C}_{S} \right)=\overline{C}_{B}-\overline{C}_{S}>0$ hold, the profit of Physician S is higher with a price cut.
2. When Physician B reduces his price, Physician S's expected benefit is 0 whatever he decreases his price or not, and it makes no difference whether physician B decreases his price or not.
3. When Physician S does not reduce his price, Physician B's dominant strategy is to reduce his price. Comparing the profits when physician B lowers his price with those when he does not:$2\left( 1-h \right)\left( \overline{C}_{B}-\underline{C}_{B} \right)-\left( \underline{C}_{S}-\underline{C}_{B} \right)>0$, or$2\left( \underline{C}_{S}-\underline{C}_{B} \right)-\left( \underline{C}_{S}-\underline{C}_{B} \right)=\underline{C}_{S}-\underline{C}_{B}>0$, the profit for Physician B is higher with a price reduction.
4. When Physician S reduces his price, Physician B's dominant strategy is to reduce his price. Clearly there is $2\left[ \left( 1-h \right)\left( \underline{C}_{S}-\underline{C}_{B} \right)+h\left( \overline{C}_{S}-\overline{C}_{B} \right) \right]>0$, Physician S will take higher profit if he lowers his price.

The analysis above shows that the dominant strategy for both Physician S and Physician B is to reduce prices, and both types of physicians will give equal markups that show their type. Therefore the market will continue to reduce prices along the Bertrand competitive path until it comes down to the cost level of Physician S who has a higher overall cost $(\underline{C}_{S}, \overline{C}_{S})$. Patients will receive honest diagnosis and treatment.

**III.** The overall costs of two types of physicians are the same, i.e.$\left( 1-h \right)\underline{C}_{S}+h\overline{C}_{S}=\left( 1-h \right)\underline{C}_{B}+h\overline{C}_{B}$.

The expected benefit for both types of physicians in each case is essentially the same as the expected benefit when the overall cost of Physician B is low. This is a standard prisoner's dilemma game that results in both physicians reducing their prices, and patients will receive honest diagnoses and treatment.

# Supplementary Figures and Tables

## Supplementary Tables

| Table A1. Non-parametric test results of patient outcomes | | | |
| --- | --- | --- | --- |
|  | Mon-Unobs  vs. Mon-Obs | Mon-Unobs  vs. Comp | Mon-Unobs  vs. Comp-Intensive |
| Cured rate | z = -16.962  P=0.0000 | z = -28.149  P=0.0000 | z = -28.924  P=0.0000 |
| Rejection rate | z=10.268  P=0.0000 | z=28.831  P=0.0000 | z=30.572  P=0.0000 |
| Rate of being honest treated | z = -15.243  P=0.000 | z = -10.311  P=0.0000 | z = -7.505  P=0.0000 |
| Rate of being overtreated | z = -6.140  P=0.0001 | z = -24.792  P=0.0000 | z = -29.397  P=0.0000 |
| Rate of being undertreated | z=13.564  P=0.0003 | z=4.957  P=0.0000 | z=-2.313  P=0.0207 |

## Supplementary Figures


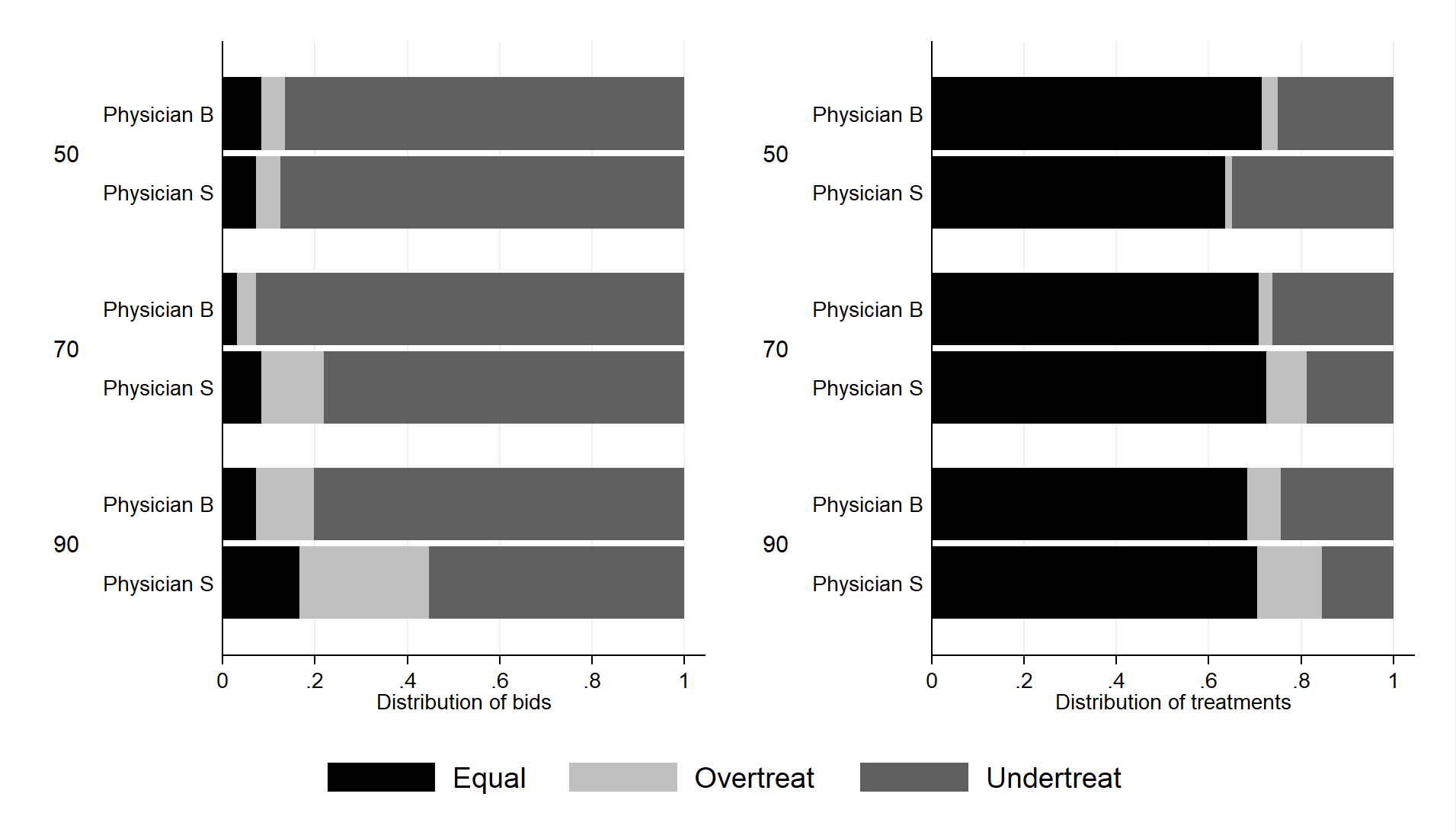


**Figure A1****. Price Type and Treatment Behavior in the *Mono-Unobs* Treatment**

Our model predicts that the physicians' bids in *Mon-Unobs* treatment are influenced by patient health gains v, and physicians’ treatment behavior will also be influenced. According to the prediction of Proposition 2, physicians will only price in regions R1, R2 and R4 when v = 50, Physician B will not overtreat his patients, and the level of overtreatment in the market is relatively low; physicians will only price in regions R2, R4 and R5 when v = 90, Physician S will not undertreat his patients, and the level of undertreatment in the market is relatively low. Figure A1. shows the distribution of biding types and treatment behavior of different types of physicians in the Mon-Unobs treatment for patients with different health gains v. The left side of Figure A1. shows the cumulative distribution of bids, with the proportion of overtreat markups for Physician B at v=50 is significantly lower than that at v=90 (Mann-Whinety test, below: z=-1.774, p=0.0761), and the proportion of undertreat markups for Physician S at v=50 is significantly lower than that at v=90 (z=-4.936, p=0.0000). The right side of Figure A1. shows the cumulative distribution of treatment behavior, and it can be observed that Physician B provides significantly less overtreatment at v=50 than that at v=90 (z=-1.738, P=0.0822), and Physician S provides significantly less undertreatment at v=90 than that at v=50 (z=-4.936, P=0.0000). However, the results above only corroborate the trend predicted by the theory, and fail to confirm the market results predicted by the theory. In Figure A1., it can be seen that in the Mon-Unobs treatment, regardless of the type of physician in any v condition, they always bid undertreat markups and cheat more often with undertreatment. Comparing the data in Figure A1. with Table 3., it shows that changes in patient health benefits v have a slight but not decisive effect on physician behavior.
